# Safety of apixaban and rivaroxaban compared to warfarin after cardiac surgery

**DOI:** 10.1111/jocs.17203

**Published:** 2022-12-07

**Authors:** Kushal D. Naik, Bryan A. Whitson, Eric M. McLaughlin, Nancy B. Matre, Alan J. Rozycki

**Affiliations:** ^1^ Department of Pharmacy Cleveland Clinic Foundation Columbus Ohio USA; ^2^ Department of Surgery The Ohio State University Wexner Medical Center Columbus USA; ^3^ Department of Biostatistics The Ohio State University Columbus Ohio USA; ^4^ Department of Quality and Operations The Ohio State University Wexner Medical Center Columbus Ohio USA; ^5^ Department of Pharmacy The Ohio State University Wexner Medical Center Columbus Ohio USA

**Keywords:** anticoagulation, cardiac surgery, DOACs, ISTH major bleeding, postoperative atrial fibrillation, warfarin

## Abstract

**Background:**

Direct oral anticoagulants (DOACs) are frequently prescribed for the management of atrial fibrillation and venous thrombosis. There is a lack of published data on the utilization of DOACs in individuals who have undergone recent cardiac surgery. The purpose of this study was to evaluate the safety and efficacy of apixaban and rivaroxaban compared to warfarin in patients postcardiac surgery.

**Methods:**

In this retrospective cohort study, patients were separated into a DOAC cohort or a warfarin cohort based on the agent they received after cardiac surgery. Patients could be included if they were ≥18 years of age and received or were discharged on either rivaroxaban, apixaban, or warfarin within 7 days after cardiac surgery. The primary outcome for the study was the rate of International Society on Thrombosis and Hemostasis (ISTH) major bleeding during hospitalization and for 30 days following discharge or until first follow‐up appointment.

**Results:**

There were a total of 194 patients included in the analysis, 97 in the DOAC cohort and 97 in the warfarin cohort. Four patients (4.1%) in the DOAC group experienced ISTH major bleeding, while 2 patients (2.1%) in the warfarin cohort experienced ISTH major bleeding (*p* = 0.68). No patients in the DOAC cohort experienced a thrombotic event, whereas 2 patients (2.1%) in the warfarin cohort experienced a thrombotic complication (*p* = 0.5).

**Conclusion:**

Apixaban and rivaroxaban demonstrated similar safety when compared to a matched cohort of warfarin patients. Larger prospective randomized studies are needed to confirm these findings.

**List of Abbreviations**
AVR: aortic valve replacement
BMI: body mass index
CABG: coronary artery bypass grafting
CKD: chronic kidney disease
COPD: chronic obstructive pulmonary disease
CVA: cerebrovascular accident
DOAC: direct oral anticoagulant
ESRD: end‐stage renal disease
ICU: intensive care unit
INR: international normalized ratio
ISTH: International Society on Thrombosis and Hemostasis
LAAL: left atrial appendage ligation
MVR: mitral valve replacement
MV repair: mitral valve repair
POAF: postoperative atrial fibrillation
POD: postoperative day
REDCap: Research Electronic Data Capture
TAVR: Transcatheter Aortic Valve Replacement
TVR: tricuspid valve replacement
VTE: venous thromboembolism

## 1 INTRODUCTION

Contemporary treatment guidelines recommend the first line use of apixaban, rivaroxaban, edoxaban, or dabigatran for stroke prophylaxis in the management of non‐valvular atrial fibrillation and the treatment of venous thrombosis (VTE).^1^, ^2^ Compared to the vitamin K antagonist, warfarin, direct oral anticoagulant (DOACs) possess the beneficial attributes of a quicker onset of action, lack of need to bridge to therapeutic anticoagulation, reduced frequency of monitoring, decreased drug−drug interactions, and minimal drug−food interactions. As a result of these positives, there is interest in the use of these agents when indicated in patients who have undergone recent cardiac surgery. On the other hand, there remains hesitancy in adopting DOACs as the standard of practice for anticoagulation in patients who have undergone recent cardiac surgery because of limitations in monitoring their efficacy, and the lack of robust data demonstrating safety of these agents in postsurgical patients who often have a high bleeding risk.

Despite the paucity of literature evaluating DOACs after cardiac surgery, there has been an increase in utilization of these agents as demonstrated in a recent query of the Society of Thoracic Surgeons (STS) Database.^3^ Current published data evaluating the utility of DOACs in postcardiac surgery patients is limited by small sample size, agents not commonly used in our clinical practice (edoxaban), and/or exclusion of common cardiac procedures such as valve replacement or repair.

Landmark studies of the major DOAC agents were not designed to study outcomes in cardiac surgery patients.^4^, ^5^ Due to the limitations with existing data, questions remain about the safety and efficacy of initiating apixaban or rivaroxaban during the index hospitalization in patients who undergo cardiac surgery. Given the growing use and preference for apixaban and rivaroxaban in the United States,^6^ the authors of this study hypothesized that apixaban and rivaroxaban would provide similar safety and efficacy to warfarin in patients who have undergone cardiac surgery.

## 2 MATERIALS AND METHODS

In this single center retrospective cohort study, patients who received apixaban or rivaroxaban were compared to those who received warfarin and underwent cardiac surgery from January 1, 2013 to October 21, 2021. This investigation was approved by local Institutional Review Board on November 15, 2021 (IRB#: 2021H0376) with waiver of need for individual consent.

Inclusion criteria comprised of patients who underwent cardiac surgery and were either discharged on or administered at least one dose of either rivaroxaban, apixaban, or warfarin within 7 days of cardiac surgery with the intention of oral anticoagulation treatment for at least 30 days. Seven days after cardiac surgery was chosen as the cutoff point to capture the high bleeding risk associated with recent surgery as well as to ensure inclusion of patients who develop postoperative atrial fibrillation (POAF), which usually occurs 2−4 after surgery^7^ with therapeutic oral anticoagulation commencing on average time 4.3 ± 2.8 days^8^ from postoperation day 0. Individuals who were pregnant, incarcerated, or had a body mass index (BMI) greater than 50 kg/m^2^ were excluded. Furthermore, patients were excluded if they received anticoagulation for the following indications: left ventricular thrombus, triple positive antiphospholipid syndrome, homozygous factor V leiden, mechanical heart valve, ventricular assist device, or Transcatheter Aortic Valve Replacement.

**Figure 1:**
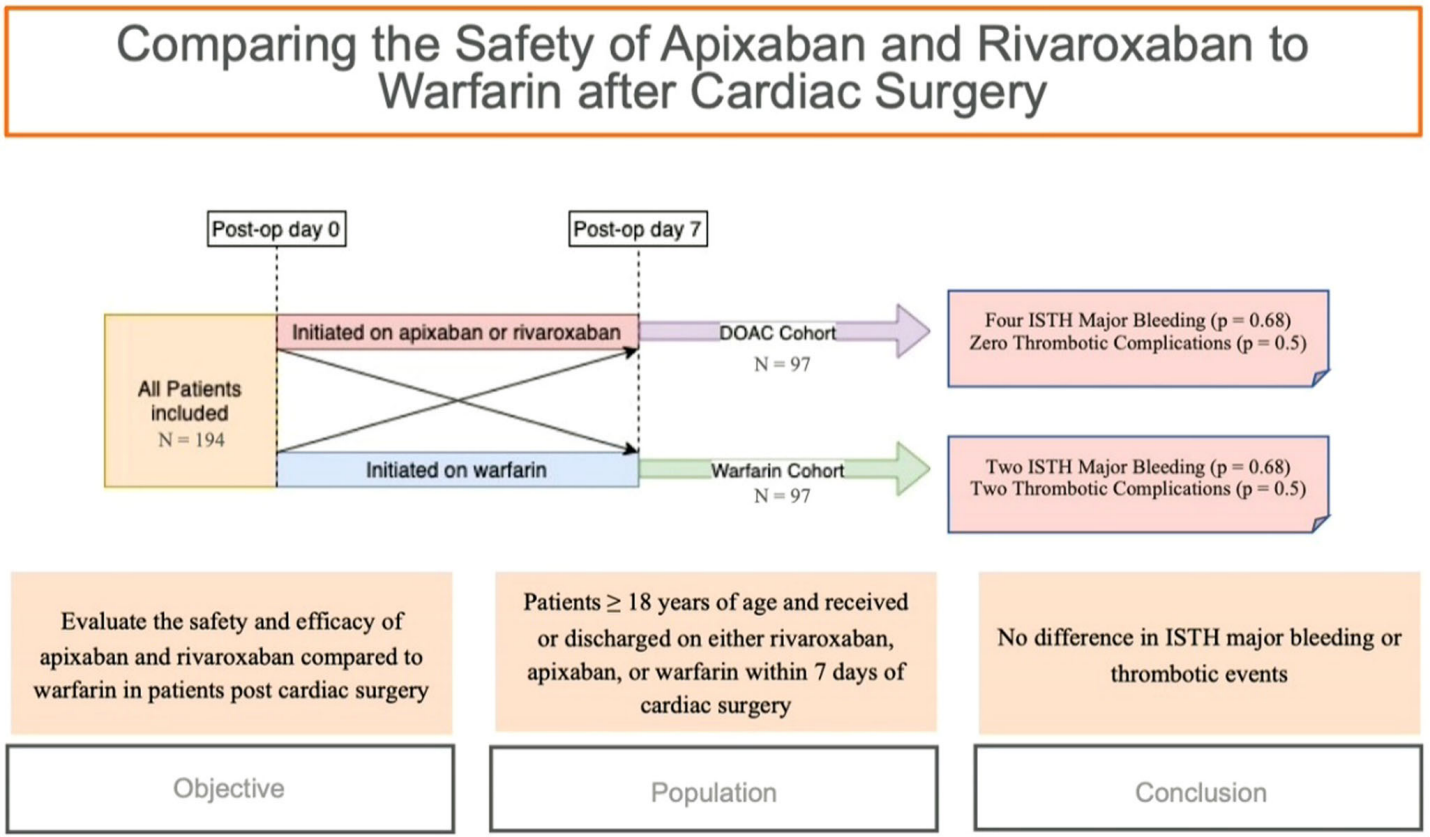
Graphical abstract—Patients who were initiated on apixaban or rivaroxaban within 7 days of cardiac surgery were assigned to the DOAC cohort. Patients initiated on warfarin within 7 days of cardiac surgery were assigned to the warfarin cohort. If a patient was initially started on a DOAC but switched to warfarin within 7 days of surgery, then they were assigned to the warfarin cohort. If a patient was started on warfarin but switched to a DOAC within 7 days of surgery, then they were placed in the DOAC group. Patients in both cohorts experienced the same total number of ISTH major bleeding and thrombotic complications. DOAC, Direct oral anticoagulants; ISTH,  International Society on Thrombosis and Hemostasis.

Patients were separated into two different cohorts based on the anticoagulant they received. Patients who received apixaban or rivaroxaban were assigned to the DOAC cohort. Apixaban and rivaroxaban were selected as the DOACs of interest since dabigatran and edoxaban are less frequently prescribed in the United States.^6^ Those who received warfarin were assigned to the warfarin cohort. If a patient was initially started on a DOAC but switched to warfarin within 7 days of surgery, then they were placed in the warfarin cohort for analysis. If a patient was started on warfarin but switched to a DOAC within 7 days of surgery, then they were placed in the DOAC group (Figure 1). To ensure patients in both cohorts had similar bleeding and thrombotic risk, patients in the warfarin cohort were matched individually to patients in the DOAC cohort based on type of cardiac surgery, anticoagulation indication, concomitant antiplatelet medications, and on versus off pump Coronary artery bypass graft (CABG) (if applicable). Concomitant surgical ablation or left atrial appendage ligation (LAAL) procedures were recorded but were not taken into consideration when matching if occurring with another primary surgical operation. Anticoagulation indication was matched based on atrial fibrillation/flutter and venous thromboembolism. Concomitant antiplatelet agents were matched based on no concomitant antiplatelet therapy, concomitant single antiplatelet therapy, and concomitant dual antiplatelet therapy. Matching based on these four classifications was used as opposed to propensity score matching to ensure that major variables associated with the risk of bleeding and thrombosis were compared between the two cohorts.

Patients were evaluated for the primary outcome of International Society on Thrombosis and Hemostasis (ISTH) major bleeding during hospitalization and for 30 days following discharge or until first follow‐up appointment. We utilized ISTH definition for bleeding as it was used in previous studies evaluating DOACs and is able to be readily be captured retrospectively. Bleeding must be related to anticoagulation use, defined as bleeding within 24 h of previous dose of a DOAC in the inpatient setting, international normalized ratio (INR) > 1.8 for warfarin patients in the inpatient setting, or bleeding that occurred in the outpatient setting. ISTH major bleeding is defined as fatal bleeding, symptomatic bleeding in a critical area or organ, bleeding causing a fall in hemoglobin level of 2‐g/dl or more, and/or bleeding leading to transfusion of two or more units of whole blood or red cells.^9^ Primary analysis occurred based on cohort assignment.

Secondary outcomes included thromboembolic events during hospitalization and for 30 days following discharge or until first follow‐up appointment, readmission within 30 days of discharge for a bleeding or thromboembolic event, time from oral anticoagulation initiation to bleeding or thromboembolic event, hospital length of stay, intensive care unit (ICU) length of stay, and mortality. Thromboembolic events were defined as systemic thromboembolism including VTE, intracardiac thrombus, or ischemic stroke.

Data was collected via manual chart review in the electronic medical record as well as procurement of historical data from the national STS database. Study data were collected and managed using Research Electronic Data Capture (REDCap) electronic data capture tools hosted at The Ohio State University.^10^, ^11^ REDCap is a secure, web‐based software platform designed to support data capture for research studies, providing (1) an intuitive interface for validated data capture; (2) audit trails for tracking data manipulation and export procedures; (3) automated export procedures for seamless data downloads to common statistical packages; and (4) procedures for data integration and interoperability with external sources.

A sample size of at least 328 patients (164 per treatment) was estimated to provide 80% power at a 5% type 1 error rate to test the non‐inferiority of DOAC treatment relative to warfarin, assuming 5% of patients in each group have the primary outcome of ISTH major bleeding with non‐inferiority defined as the outcome proportion in the DOAC group of no more than 6% greater than the outcome proportion in the warfarin group. We chose a non‐inferiority margin of 6% as bleeding rates with in patients receiving DOACs after cardiac surgery is not established and we deemed a 6% increase as clinically significant. A one‐sided *t*‐test was used to determine statistical power.

Summary statistics were calculated, and continuous variables were reported as means (standard deviations) or medians [first‐third quartiles] where relevant. Categorical variables were reported as frequencies (percentage). Hypothesis testing comparing treatment groups was conducted using Student's *t*‐tests or Wilcoxon rank sum tests for continuous variables and chi‐square or Fisher's exact tests where relevant. A test for noninferiority of DOAC to warfarin was performed using a one‐sided Z‐test with a margin of −6%. *p* < 0.05 was considered statistically significant. All analyses were conducted using SAS 9.4 (SAS Institute).

## 3 RESULTS

**Figure 2:**
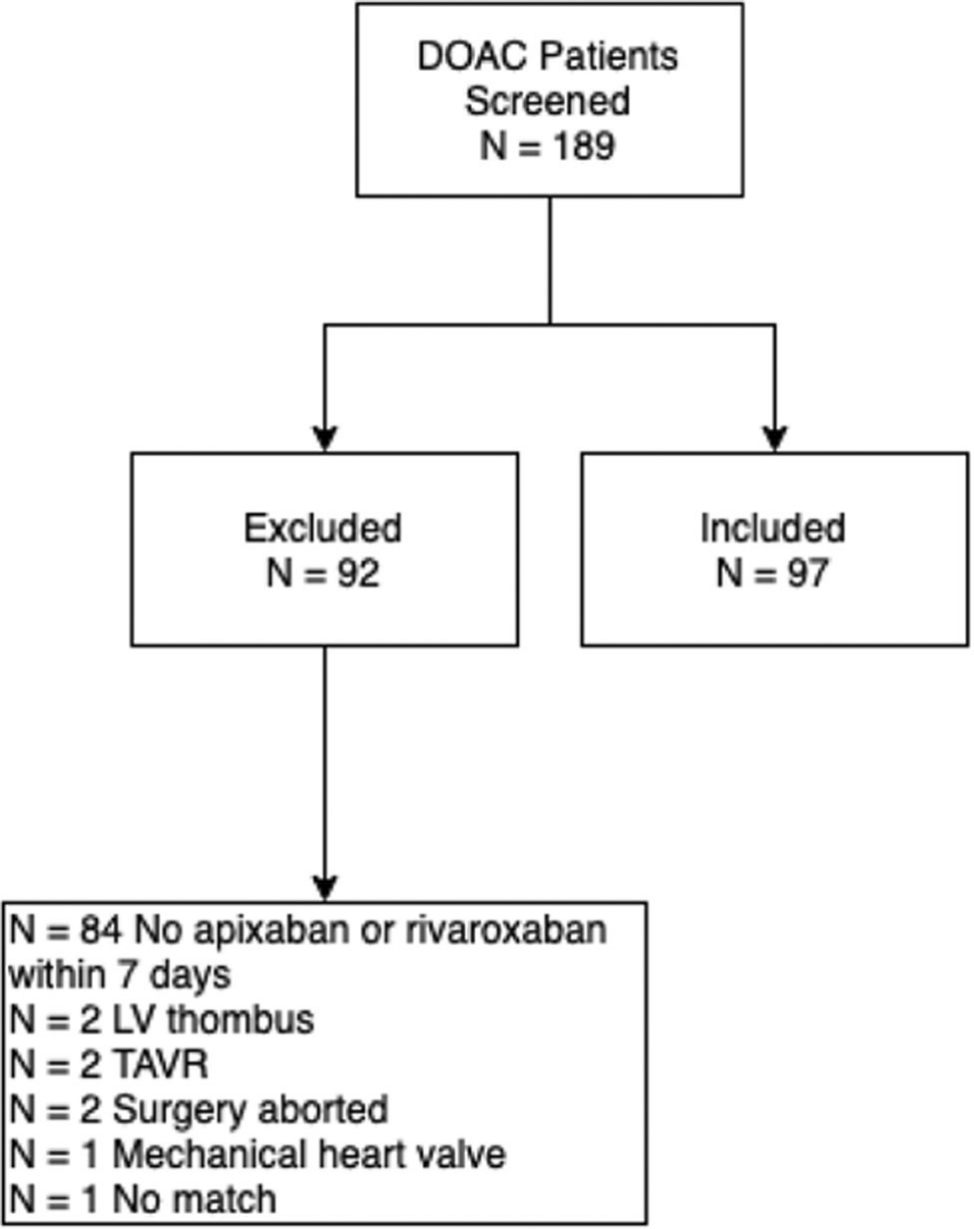
Of the 189 patients screened for inclusion, 97 were included and 92 were excluded. The most common reason for exclusion from the study was no initiation of apixaban or rivaroxaban within 7 days.

There were 189 DOAC patients who were screened, and 97 which met inclusion criteria (Figure 2). The most common reason for exclusion from the study was because the oral anticoagulant was not initiated within 7 days of cardiac surgery. A list of 6,186 warfarin patients form the same time period was used to screen for patients that met the inclusion criteria and who could be individually matched to a DOAC patient. Therefore, 97 warfarin patents were matched to the included DOAC patients to make up a total study population of 194 patients. The median age in the DOAC cohort was 63.7 years [IQR 55.5−70.3], while median age in the warfarin cohort was 67.1 years [IQR 59.3−74.3] (*p* = 0.008). There was no significant difference in the cohorts with respect to percentage of females, BMI, or anticoagulation use before surgery (Table 1). More patients in the warfarin cohort had CKD stage 3 or higher (14.4% vs. 2.1%; *p* = 0.003). The most common indication for anticoagulation after cardiac surgery was atrial fibrillation/atrial flutter (85.6%). Apixaban was prescribed as the DOAC in 67 (69.1%) patients from the DOAC cohort (Table 2).

**Table 1:** Population characteristics

Variables	DOAC	Warfarin	*p* Value
	*N* = 97	*N* = 97	
Female, *n* (%)	27 (27.8)	25 (25.8)	0.87
Age at time of surgery, median years [IQR]	63.7 [55.5−70.3]	67.1 [59.3−74.3]	0.008
Body mass index, median kg/m^2^ [IQR]	29.4 [26.9−35.7]	29.9 [27.0−35.0]	0.91
ICU length of stay, median hours [IQR]	36.6 [34.6−58.9]	50.8 [34.7−105.6]	0.06
Postoperative length of stay, median days [IQR]	6 [5–7]	8 [6–11]	<0.001
Total length of stay, median days [IQR]	7 [5–10]	10 [7–15]	<0.001
Comorbidities			
Hypertension, *n* (%)	67 (69.1)	79 (81.4)	0.07
Previous documented bleeding, *n* (%)	5 (5.2)	8 (8.3)	0.57
History of CVA/stroke, *n* (%)	4 (4.1)	10 (10.3)	0.16
CKD3 or below, *n* (%)	2 (2.1)	14 (14.4)	0.003
Diabetes, *n* (%)	22 (22.7)	17 (17.5)	0.47
Anticoagulation use before surgery			
Yes, *n* (%)	70 (72.2)	65 (67.0)	0.53
Indication for anticoagulation before surgery			
Atrial Fibrillation/Flutter, *n* (%)	60 (61.9)	56 (57.7)	0.66
DVT/PE, *n* (%)	11 (11.3)	10 (10.3)	>0.99
Indication for anticoagulation after surgery			
Atrial fibrillation/flutter, *n* (%)	83 (85.6)	83 (85.6)	>0.99
VTE, *n* (%)	14 (14.4)	14 (14.4)	>0.99

Abbreviations: CVA, cerebrovascular accident; DOAC, Direct oral anticoagulants; DVT, deep vein thrombosis; ICU, intensive care unit; IQR, interquartile range; PE, pulmonary embolism; VTE, treatment of venous thrombosis.

**Table 2:** Distribution of agents in DOAC cohort

Anticoagulant after cardiac surgery		DOAC
		*N* = 97
Apixaban, *n* (%)		67 (69.1)
Rivaroxaban, *n* (%)		30 (30.9)

Abbreviation: DOAC, Direct oral anticoagulants.

The most frequent cardiac operations were CABG (34%) and surgical ablations alone (26.8%), respectively (Table 3). There was no difference in concomitant LAAL procedures performed (39.4% vs. 32.4%; *p* = 0.76) or concomitant atrial fibrillation ablation (21.1% vs 25.4%, *p* = 0.36) in the DOAC or warfarin cohorts, respectively. There was no significant difference in the primary outcome of ISTH major bleeding between the DOAC and warfarin cohorts (4.1% vs. 2.1%, *p* = 0.68; Table 4). When the noninferiority of DOACs was tested, with a margin of up to a 6% higher rate of ISTH major bleeding relative to warfarin, the result was not statically significant (*p* = 0.06). All 4 patients in the DOAC cohort who experienced ISTH major bleeding received apixaban (Table 5). Of the 4 DOAC patients who had an ISTH major bleeding event, all had undergone CABG surgery (3 on‐pump, 1 off‐pump). Of the 2 patients in the warfarin cohort who experienced ISTH major bleeding, 1 had undergone on‐pump CABG, while the other had a bioprosthetic aortic valve replacement. One of the warfarin patients who experienced ISTH major bleeding had a history of end stage renal disease. The indication for anticoagulation was atrial fibrillation/flutter in all patients who had ISTH major bleeding. None of the patients who experienced major bleeding were prescribed concomitant dual antiplatelet therapy (Table 5). The median duration of anticoagulation until a bleeding event occurred was 7 days [IQR 5−10]. Of the six patients who had a bleeding event, five were readmitted to the hospital within 30 days of discharge, whereas one patient experienced the bleeding event during their hospital stay. There were no significant differences in immediate postoperative antithrombotic characteristics between the two cohorts as it relates to aspirin dose or exposure to unfractionated heparin or low‐molecular weight heparin (Supporting Information: Table 1).

**Table 3:** Type of cardiac surgery

Type of surgery			DOAC	Warfarin	*p* Value
			*N* = 97	*N* = 97	
Ascending aortic replacement/repair, *n* (%)			6 (6.2)	6 (6.2)	>0.99
CABG, *n* (%)			33 (34.0)	33 (34.0)	>0.99
On‐pump CABG, *n* (%)			8 (8.3)	8 (8.3)	>0.99
Combination CABG and valve, *n* (%)			3 (3.1)	3 (3.1)	>0.99
AVR, *n* (%)			7 (7.2)	7 (7.2)	>0.99
MV repair/MV replacement, *n* (%)			10 (10.3)	10 (10.3)	>0.99
Pulmonary thromboendarterectomy, *n* (%)			6 (6.2)	6 (6.2)	>0.99
Surgical ablation, *n* (%)			26 (26.8)	26 (26.8)	>0.99
Combination valve repair, *n* (%)			3 (3.1)	3 (3.1)	>0.99
Other, *n* (%)			3 (3.1)	6 (3.1)	>0.99
Concomitant procedures			***N*** = **71**	***N*** = **71**	
Atrial appendage ligation, *n* (%)			28 (39.4%)	23 (32.4%)	0.48
Atrial fibrillation ablation, *n* (%)			15 (21.1%)	18 (25.4%)	0.69

Abbreviations: AVR, aortic valve replacement; CABG, Coronary artery bypass graft; DOAC, Direct oral anticoagulants; MV, mitral valve.

**Table 4:** Outcomes

Outcome		DOAC	Warfarin	*p* Value
		*N* = 97	*N* = 97	
ISTH major bleeding, *n* (%)		4 (4.1)	2 (2.1)	0.68
Symptomatic bleeding in a critical area or organ, *n* (%)		3 (3.1)	2 (2.1)	>0.99
Bleeding leading to transfusion of two or more units of whole blood or red cells, *n* (%)		1 (1.0)	2 (2.1)	>0.99
Rehospitalization for bleeding, *n* (%)		4 (4.1)	1 (1.0)	0.37
Any rehospitalization, *n* (%)		13 (13.4)	12 (12.4)	>0.99
Thromboembolic event, *n* (%)		0 (0)	2 (2.1)	0.50
Duration of anticoagulation until bleeding event, median days [IQR]		7 [4‐9.5]	8.5 [5–12]	0.64
Duration of anticoagulation until thrombotic event, median days [IQR]		n/a	11.5 [5–18]	n/a

Abbreviations: IQR, interquartile range; ISTH,  International Society on Thrombosis and Hemostasis.

**Table 5:** Comparison of bleeding outcome occurrences by patient factors

Variable		ISTH major bleeding	No ISTH major bleeding
		(*N* = 6)	(*N* = 188)
CHA_2_DS_2_VASC Score, median [IQR]		3 [3−4]	3 [2−4]
HASBLED Score, median [IQR]		3 [3]	2 [2−3]
Triple antithrombotic therapy, *n* (%)		0 (0)	20 (100)
Baseline CrCL < 50 ml/min, *n* (%)			
<50 ml/min, *n* (%)		0 (0)	22 (100)
ESRD, *n* (%)		1 (100)	0 (0)
Previous documented bleeding			
Yes, *n* (%)		0 (0)	13 (100)
Anticoagulant			
Warfarin, *n* (%)		2 (2.1)	95 (97.9)
Apixaban, *n* (%)		4 (6.0)	63 (94.0)
Rivaroxaban, *n* (%)		0 (0)	30 (100)

Abbreviations: ESRD, end‐stage renal disease; ISTH,  International Society on Thrombosis and Hemostasis.

No thrombotic events occurred in the DOAC cohort, while 2 thromboembolic events occurred in the warfarin cohort (Table 4). The median duration of anticoagulation until a thromboembolic event was 11.5 days [IQR 5–18] in these two patients. One patient who experienced a thrombotic event had undergone on‐pump CABG surgery and was hospitalized with an ischemic stroke. The second patient had undergone a pulmonary thromboendarterectomy and returned with a new pulmonary embolism. Rehospitalization within 30 days from discharge for any cause was similar in both cohorts (13.4% in DOAC cohort vs. 12.4% in warfarin cohort; *p* > 0.99). There was no difference in rehospitalization for a bleeding or thrombotic event or its individual components within 30‐days of discharge. Median postoperative length of stay was significantly shorter in the DOAC cohort compared to warfarin (6 days vs. 8 days, *p* = <0.001; Table 1). Likewise, the median total length of stay was also shorter in the DOAC patients compared to the warfarin group (7 days vs. 10 days, *p* = <0.001; Table 1). There was no mortality in either cohort during the hospitalization or within 30‐days of discharge.

A subgroup analysis of the individual DOAC agents demonstrated that patients who received rivaroxaban underwent surgical ablation more often than those who received apixaban (Table 6). On the other hand, patients who received apixaban underwent CABG surgery more often. Additionally, patients who received rivaroxaban had a median CHA_2_DS_2_VASC score of 2 [IQR 1‐3], while patients who received apixaban and warfarin had a median CHA_2_DS_2_VASC score of 3 [IQR 2−4] (*p* = 0.03; Table 6).

**Table 6:** Comparison of patient characteristics by anticoagulant

Variable		Apixaban	Rivaroxaban	Warfarin	*p* Value
		*N* = 67	*N* = 30	*N* = 97	
Baseline CrCL					>0.99
<50 ml/min, *n* (%)		3 (4.5)	1 (3.3)	18 (18.6)	
ESRD, *n* (%)		0 (0)	0 (0)	1 (1)	
CHA_2_DS_2_VASC score, median [IQR]		(*n* = 56)	(*n* = 27)	(*n* = 83)	0.03
		3 [2–4]	2 [1–3]	3 [2–4]	
HASBLED score, median [IQR]		(*n* = 56)	(*n* = 27)	(*n* = 83)	0.01
		2 [2−3]	2 [1–3]	3 [2–3]	
Comorbidities					
Hypertension, *n* (%)		48 (71.6)	19 (63.3)	79 (81.4)	0.09
Previous documented bleeding, *n* (%)		2 (3.0)	3 (10.0)	8 (8.3)	0.27
CVA/stroke, *n* (%)		3 (4.5)	1 (3.3)	10 (10.3)	0.35
CKD3 or below, *n* (%)		2 (3.0)	0 (0)	14 (14.4)	0.006
Diabetes, *n* (%)		16 (23.9)	6 (20.0)	17 (17.5)	0.60
Type of surgery					
CABG only, *n* (%)		28 (41.8)	5 (16.7)	33 (34.0)	0.051
AVR only, *n* (%)		6 (9.0)	1 (3.3)	7 (7.2)	0.65
CABG + valve, *n* (%)		2 (3.0)	0 (0)	2 (2.1)	1.0
Pulmonary thromboendarterectomy, *n* (%)		5 (7.5)	1 (3.3)	6 (6.2)	0.85
Surgical ablation, *n* (%)		9 (13.4)	17 (56.7)	26 (26.8)	<0.001
Days to initiation of anticoagulant from surgery, median [IQR]		5 [4–6]	4 [3–5]	3 [2–5]	<0.001

Abbreviations: AVR, aortic valve replacement; CVA, cerebrovascular accident; ESRD, end‐stage renal disease.

## 4 DISCUSSION

This retrospective cohort study of apixaban and rivaroxaban compared to warfarin in patients who underwent recent cardiac surgery demonstrated that there was no significant difference in ISTH major bleeding or thromboembolic events. The baseline characteristics in the two cohorts were generally similar except for more CKD stage 3 or greater in the warfarin cohort. This difference is not surprising given the study period started in 2013 where DOAC use in patients with renal dysfunction was less established. It should be noted that CKD is a potential risk factor for bleeding and may confound difference in bleeding rates. On the other hand, it is important to include these patients, especially with increasing use of DOAC agents in CKD patients. The net clinical benefit was equal with a total of 4 bleeding and thromboembolic events in each cohort. Rehospitalization for any cause, bleeding, or thromboembolic events were similar in each cohort. Most of the events occurred early after discharge for patients, likely from the risk of bleeding and thromboembolism from the recent cardiac surgery procedure. All of the patients who experienced bleeding events in the DOAC cohort were treated with blood products. Andexanet alfa was not utilized to reverse the DOAC agent in any of the patients in the study as this medication is non‐formulary at our institution. While all 4 patients in the DOAC cohort that experienced major bleeding were on apixaban, the interpretation should be with caution as patients who received rivaroxaban underwent surgical ablations more frequently (see Supporting Information: Table 2 for complete list of cardiac surgery type categorized by anticoagulant agent). Previous studies illustrate that surgical ablation procedures are associated with a less risk of bleeding compared to other cardiac surgeries.^12^, ^13^ The most common procedure in patients who experienced a bleeding event was CABG. While CABG can be associated with a high bleeding risk, the reason that patients who had a bleeding event were more likely to have a CABG procedure may be due to the observation that CABG was the most prevalent type of cardiac surgery in the study population. There was a significantly shorter postoperative and total length of stay in the DOAC group compared to warfarin. The ICU length of stay was similar in both cohorts. The shorter length of stay in the DOAC cohort could be due to either a less complex postoperative course in the DOAC patients or the additional time needed to achieve a therapeutic INR. A delay in discharge waiting for a therapeutic INR prior occurred in 6 (6.2%) warfarin patients as determined by retrospective chart review.

An important aspect of this study was the individual matching conducted to ensure that both cohorts were well‐balanced. By thoroughly matching the patients, we ensured that both cohorts had the same procedure types, anticoagulation indication, and concomitant antiplatelet use. This strategy minimized confounding variables and allowed for interpretation of the drug effects on safety and efficacy.

The first publication evaluating the use of DOACs in patients after cardiac surgery was conducted by Anderson et al.^7^ In this single center retrospective study, 27 patients who developed POAF after CABG were discharged on apixaban, dabigatran, or rivaroxaban compared to 45 patients who were discharged on warfarin. The study demonstrated no increase in major bleeding for patients receiving DOACs. Our study demonstrated similar results in patients who underwent a variety of types of cardiac surgery procedures.

A previous study by Sezai et al compared bleeding rates in 135 patients who received edoxaban, apixaban, or rivaroxaban for POAF after cardiac surgery found overall bleeding rates to be low, however rivaroxaban was associated with a significantly higher bleeding rate compared to apixaban or edoxaban.^14^ Our finding of less bleeding events in the rivaroxaban subgroup differs from these results. The difference can likely be explained by the lower bleeding risk procedures that patients underwent in the rivaroxaban subgroup. In the study by Sezai et al., more than 60% of patients were on edoxaban, which is less frequently prescribed in the United States. Insurance claims data have shown that of all the DOACs, edoxaban is prescribed less than 1% of time in the United States.^6^

This study has several limitations. The retrospective design exposes the analysis to selection bias and inability to control for confounding factors. The study was also underpowered to test the intended non‐inferiority margin. Patients who are able to start oral anticoagulation within 7 days of surgery likely have less postoperative complications, thus the data may be less applicable to more complex patients. Further, major outcomes captured are short‐term, thus limiting the ability to draw conclusions about long‐term safety and efficacy. While this is one of the larger studies evaluating DOACs in recent cardiac surgery patients, the sample size is still small and did not achieve power to demonstrate the non‐inferiority of DOACs.

Despite these limitations, there were important strengths to highlight about this study. Previous studies had either excluded patients who had undergone procedures other than CABG or indications for anticoagulation outside of POAF. This study included all procedures performed and multiple indications for anticoagulation. Therefore, this methodology allowed our study to be more pragmatic than previous literature. In addition, this is the first study matching patients in the different cohorts. We matched on type of procedure, concomitant antiplatelets, indications of anticoagulation, and on vs off pump for CABG procedures (Supporting Information: Table 3). Using this approach allowed important confounders to be controlled for that could predispose patients to bleeding or thrombosis. Furthermore, in this study all patients were started on an oral anticoagulant during the index hospitalization of cardiac surgery and within 7 days of the cardiac procedure. The RIVER trial investigated whether rivaroxaban was non‐inferior to warfarin in patients with atrial fibrillation and bioprosthetic mitral valves.^15^ While the results were promising and showed non‐inferiority of rivaroxaban to warfarin, only 18% of patients were included within 3 months of their cardiac surgery and it is unknown how many patients were initiated on rivaroxaban during the index hospitalization for cardiac surgery. This study evaluated the bleeding risk of apixaban and rivaroxaban, which are the most commonly prescribed DOACs in the United States,^14^ compared to warfarin in a recent cardiac surgery patient population.

## 5 CONCLUSION

This study is one of the largest to date evaluating the safety of DOACs in patients who have undergone recent cardiac surgery. This result provides information of the effectiveness of these medications in routine clinical practice for patients who had undergone different types of common cardiac surgeries.

In conclusions, this study compared the most commonly used DOACs in the United States, apixaban and rivaroxaban, to warfarin in patients who had undergone recent cardiac surgery. There was no statistically significant difference in ISTH major bleeding or thromboembolic events between the two groups. The promising results of this study highlight the need for a large prospective randomized clinical trial to definitively make conclusions about the safety of apixaban and rivaroxaban when compared to warfarin in patients who have undergone recent cardiac surgery.

## AUTHOR CONTRIBUTIONS

**Kushal D. Naik**: Concept/design, data collection, data analysis/interpretation, drafting article, and approval of article. **Bryan Whitson**: data analysis/interpretation, critical revision of article, and approval of article. **Eric McLaughlin**: statistics, data analysis/interpretation, and approval of article. **Nancy Matre**: data collection and approval of article. **Alan Rozycki**: concept/design, data collection, data analysis/interpretation, critical revision of article, and approval of article.

## CONFLICT OF INTEREST

The authors declare no conflict of interest.

## ETHICS STATEMENT

This investigation was approved by local Institutional Review Board on 11/15/2021 (IRB#: 2021H0376) with waiver of need for individual consent.

## Supporting information

Supporting information.
