# Supplementary material for: Safety of apixaban and rivaroxaban compared to warfarin after cardiac surgery
Source: J Card Surg. 2022 Dec 7;37(12):4740–7. doi: 10.1111/jocs.17203 (PMC10107629; doi:10.1111/jocs.17203)
Supplement: Supplementary file 1 — Supporting information. [file JOCS-37-4740-s001.docx]

Supplementary Material:

**Supplement Table 1. Postoperative Antithrombotic Characteristics**

| **Variable** | | **All Patients**  **N=194** | **DOAC**  **N=97** | **Warfarin**  **N=97** | **P-value** |
| --- | --- | --- | --- | --- | --- |
| **Heparin or enoxaparin post-op** | |  |  |  |  |
|  | Yes, *n* (%) | 178 (91.8) | 85 (87.6) | 93 (95.9) | 0.06 |
|  | No, *n* (%) | 16 (8.3) | 12 (12.4) | 4 (4.1) |  |
| **Type of Heparin or enoxaparin** | |  |  |  | 0.007 |
|  | IV heparin, *n* (%) | 95 (53.4) | 36 (42.4) | 59 (63.4) |  |
|  | DVT prophylaxis heparin, *n* (%) | 70 (39.3) | 39 (45.9) | 31 (33.3) |  |
|  | DVT prophylaxis enoxaparin, *n* (%) | 13 (7.3) | 10 (11.8) | 3 (3.2) |  |
| **Change from one anticoagulant to another** | |  |  |  | n/a |
|  | No change, *n* (%) | 187 (96.4) | 90 (92.8) | 97 (100) |  |
|  | warfarin to apixaban, *n* (%) | 4 (2.1) | 4 (4.1) | 0 (0) |  |
|  | warfarin to rivaroxaban, *n* (%) | 3 (1.5) | 3 (3.1) | 0 (0) |  |
|  | apixaban to warfarin, *n* (%) | 0 (0) | 0 (0) | 0 (0) |  |
|  | rivaroxaban to warfarin, *n* (%) | 0 (0) | 0 (0) | 0 (0) |  |
| **Antiplatelets** | |  |  |  |  |
|  | Aspirin 81 mg, *n* (%) | 164 (97.0) | 79 (81.4) | 85 (87.6) | 0.32 |
|  | Aspirin 325 mg, *n* (%) | 5 (3.0) | 5 (5.2) | 0 (0) | 0.06 |
|  | Clopidogrel, *n* (%) | 23 (11.9) | 12 (12.4) | 11 (11.3) | 1.0 |

**Supplement Table 2. Comparison of patient characteristics by anticoagulant**

| **Variable** | | **Apixaban**  **N=67** | **Rivaroxaban**  **N=30** | **Warfarin**  **N=97** | **P-value** |
| --- | --- | --- | --- | --- | --- |
| **CHA2DS2-VASC score**, median [IQR] | | (n=56)  3 [2-4] | (n=27)  2 [1-3] | (n=83)  3 [2-4] | 0.03 |
| **HASBLED score**, median [IQR] | | (n=56)  2 [2-3] | (n=27)  2 [1-3] | (n=83)  3 [2-3] | 0.01 |
| **Comorbidities** | |  |  |  |  |
|  | Hypertension, *n* (%) | 48 (71.6) | 19 (63.3) | 79 (81.4) | 0.09 |
|  | Previous documented bleeding, *n* (%) | 2 (3.0) | 3 (10.0) | 8 (8.3) | 0.27 |
|  | CVA/stroke, *n* (%) | 3 (4.5) | 1 (3.3) | 10 (10.3) | 0.35 |
|  | CKD3 or below, *n* (%) | 2 (3.0) | 0 (0) | 14 (14.4) | 0.006 |
|  | COPD, *n* (%) | 18 (26.9) | 3 (10.0) | 19 (19.6) | 0.16 |
|  | Diabetes, *n* (%) | 16 (23.9) | 6 (20.0) | 17 (17.5) | 0.60 |
|  | Hypercoagulable state, *n* (%) | 8 (11.9) | 5 (16.7) | 10 (10.3) | 0.60 |
| **Type of Surgery** | |  |  |  | n/a |
|  | Ascending aortic , *n* (%) | 3 (4.5) | 0 (0) | 3 (3.1) |  |
|  | Ascending aortic + AVR, *n* (%) | 1 (1.5) | 1 (3.3) | 2 (2.1) |  |
|  | Ascending aortic + CABG, *n* (%) | 1 (1.5) | 0 (0) | 1 (1.0) |  |
|  | AVR Only, *n* (%) | 6 (9.0) | 1 (3.3) | 7 (7.2) |  |
|  | CABG Only, *n* (%) | 28 (41.8) | 5 (16.7) | 33 (34.0) |  |
|  | CABG + AVR, *n* (%) | 0 (0) | 1 (3.3) | 1 (1.0) |  |
|  | CABG + MVR, *n* (%) | 2 (3.0) | 0 (0) | 2 (2.1) |  |
|  | Intracardiac tumor removal/biopsy, *n* (%) | 1 (1.5) | 0 (0) | 1 (1.0) |  |
|  | MV repair, *n* (%) | 5 (7.5) | 2 (6.7) | 7 (7.2) |  |
|  | MV repair + TV repair, *n* (%) | 2 (3.0) | 1 (3.3) | 3 (3.1) |  |
|  | MVR Only, *n* (%) | 2 (3.0) | 1 (3.3) | 3 (3.1) |  |
|  | Pulmonary thromboendarterectomy, *n* (%) | 5 (7.5) | 1 (3.3) | 6 (6.2) |  |
|  | Resection of anomalous coronary artery fistulae, *n* (%) | 1 (1.5) | 0 (0) | 1 (1.0) |  |
|  | Surgical ablation, *n* (%) | 9 (13.4) | 17 (56.7) | 26 (26.8) |  |
|  | TVR only, *n* (%) | 1 (1.5) | 0 (0) | 1 (1.0) |  |
| **Days to initiation of anticoagulant from surgery**, median [IQR] | | 5 [4-6] | 4 [3-5] | 3 [2-5] | <0.001 |
| **Time to therapeutic INR**, median [IQR] | | N/A | N/A | (n=55)  4 [3-5] | n/a |

**Supplement Table 3. Types of Cardiac Surgery**

| **Variable** | | **All Patients**  **N=194** | **DOAC**  **N=97** | **Warfarin**  **N=97** | **P-value** |
| --- | --- | --- | --- | --- | --- |
| **Type of Surgery** | |  |  |  | n/a |
|  | Ascending aortic, *n* (%) | 6 (3.1) | 3 (3.1) | 3 (3.1) |  |
|  | Ascending aortic + AVR, *n* (%) | 4 (2.1) | 2 (2.1) | 2 (2.1) |  |
|  | Ascending aortic + CABG, *n* (%) | 2 (1.0) | 1 (1.0) | 1 (1.0) |  |
|  | AVR only, *n* (%) | 14 (7.2) | 7 (7.2) | 7 (7.2) |  |
|  | CABG only, *n* (%) | 66 (34.0) | 33 (34.0) | 33 (34.0) |  |
|  | CABG + AVR, *n* (%) | 2 (1.0) | 1 (1.0) | 1 (1.0) |  |
|  | CABG + MVR, *n* (%) | 4 (2.1) | 2 (2.1) | 2 (2.1) |  |
|  | Intracardiac tumor removal/biopsy, *n* (%) | 2 (1.0) | 1 (1.0) | 1 (1.0) |  |
|  | MV repair, *n* (%) | 14 (7.2) | 7 (7.2) | 7 (7.2) |  |
|  | MV repair + TV repair, *n* (%) | 6 (3.1) | 3 (3.1) | 3 (3.1) |  |
|  | MVR only, *n* (%) | 6 (3.1) | 3 (3.1) | 3 (3.1) |  |
|  | Pulmonary thromboendarterectomy, *n* (%) | 12 (6.2) | 6 (6.2) | 6 (6.2) |  |
|  | Resection of anomalous coronary artery fistulae, *n* (%) | 2 (1.0) | 1 (1.0) | 1 (1.0) |  |
|  | Surgical ablation, *n* (%) | 52 (26.8) | 26 (26.8) | 26 (26.8) |  |
|  | TVR only, *n* (%) | 2 (1.0) | 1 (1.0) | 1 (1.0) |  |
| **On pump vs. off pump CABG** | |  |  |  | 1.0 |
|  | On pump, *n* (%) | 58 (29.9) | 29 (29.9) | 29 (29.9) |  |
|  | Off pump, *n* (%) | 16 (8.3) | 8 (8.3) | 8 (8.3) |  |
